# Supplementary material for: Direct Detection of Antibacterial-Producing Soil Isolates Utilizing a Novel High-Throughput Screening Assay
Source: Microorganisms. 2022 Nov 11;10(11):2235. doi: 10.3390/microorganisms10112235 (PMC9698797; doi:10.3390/microorganisms10112235)
Supplement: Supplementary file 1 [file microorganisms-10-02235-s001.zip › microorganisms-1994224-supplementary.pdf]

microorganisms

MDPI

Supplementary data

Direct detection of antibacterial producing soil isolates utilizing a novel high-throughput screening assay

Wikus Ernst Laubscher [1] and Marina Rautenbach [1,*]

[1] BioPep Peptide Group, Department of Biochemistry, University of Stellenbosch, Stellenbosch, 7600, South Africa; mra@sun.ac.za

* Correspondence: mra@sun.ac.za (MR)

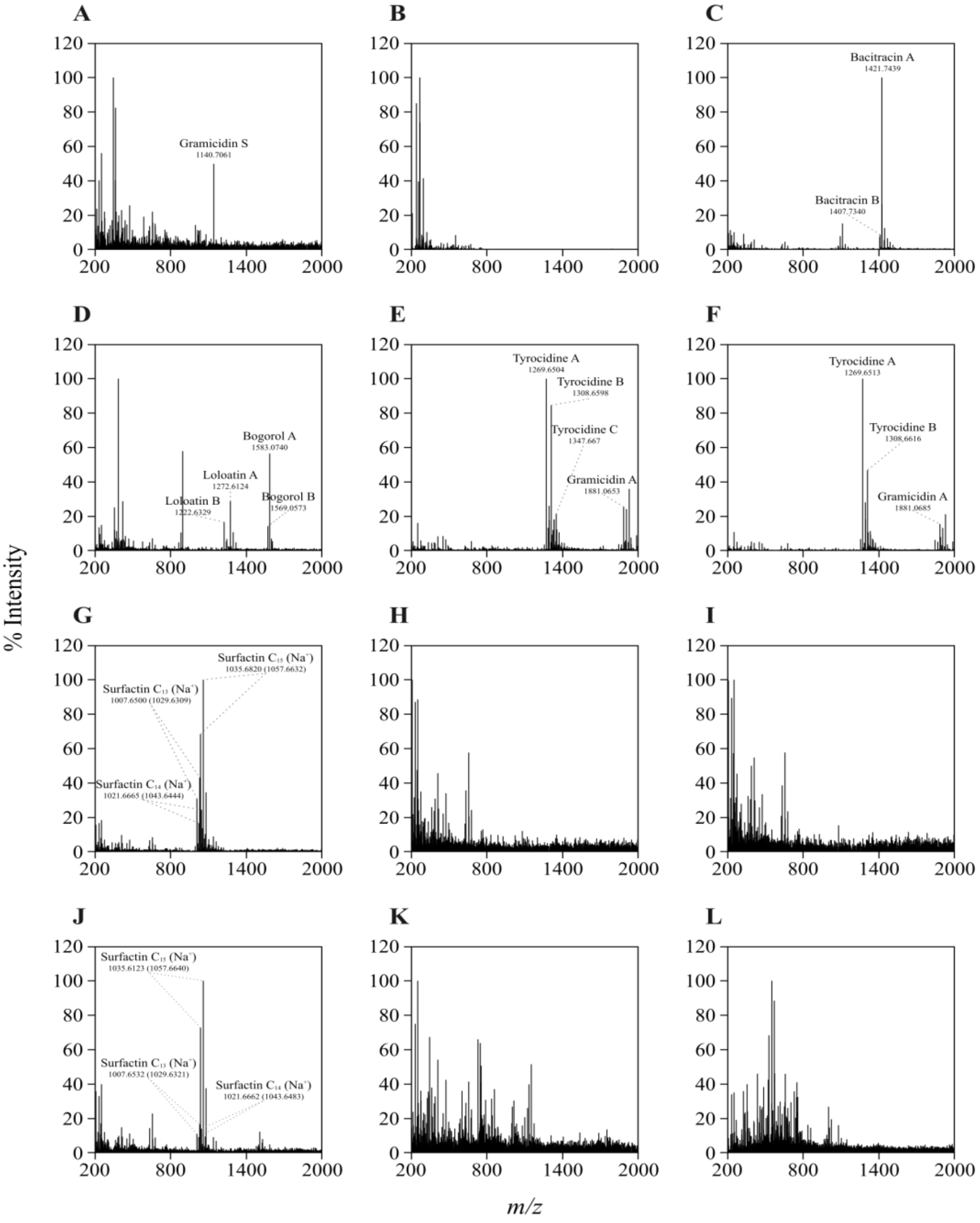

Figure S1. Mass spectra of (A) *A. migulanus* ATCC9999, (B) *P. aeruginosa* ATCC27853, (C) *B. licheniformis* LB5, (D) *Br. laterosporus* LB4, (E) *Br. parabrevis* ATCC10068, (F) *Br. parabrevis* ATCC8185, (G) *B. subtilis* OKB105, (H) *B. subtilis* OKB120, (I) *B. subtilis* OKB168, (J) *B. subtilis* ATCC21332, (K) *M. luteus* NCTC8340, (L) *E. coli* K12 extracts. Bacteria were grown on LB agar in 96 well microtiter plates at 30 °C for 7 days before being extracted with 200 µL methanol. Extracts were subjected to direct injection electron spray mass spectrometry on a Waters Synapt G2 mass spectrometer. The cone and capillary voltage of the ionization source was set to 15 V and 2.5 kV respectively. Nitrogen was used as desolvation gas at 650 L/hour and the desolvation temperature was set to 275 °C. Extracts (3 µL) were injected and eluted in the absence of a column using an isocratic gradient of 1:1 acetonitrile and 0.1% formic at 0.25 mL/min. Data was collected for 1 minute by scanning over a *m/z* range of 200 to 2000 in positive mode. All spectra per injection were combined in the MassLynx V4.1 software and deconvoluted using the MaxEnt3 algorithm included in the MassLynx V4.1 software.

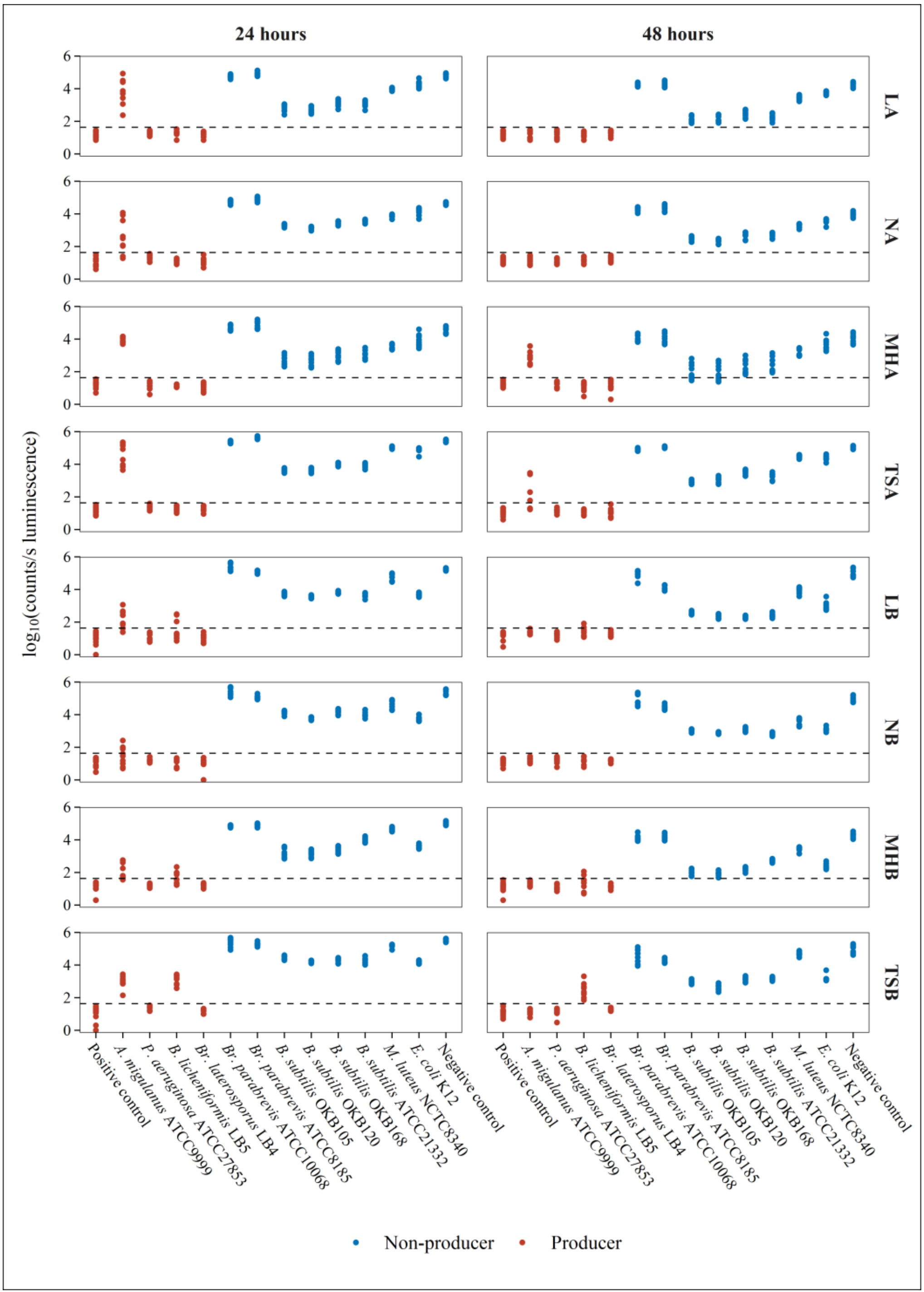

Figure S2. Luminescence of the reporter bacterium *E. coli* Xen14 in various reporter media following 24 and 48 hours of incubation with several test bacteria

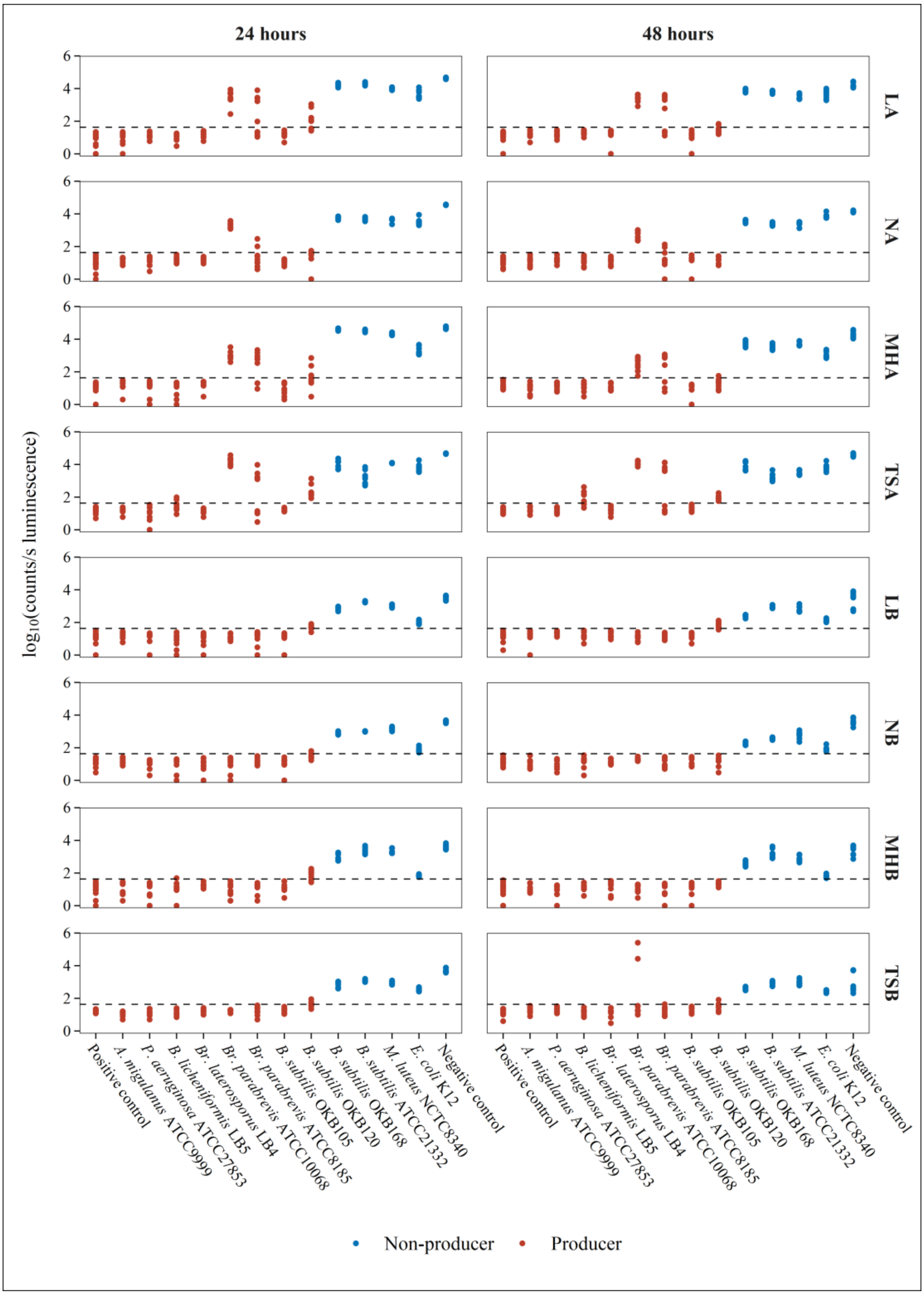

Figure S3. Luminescence of the reporter bacterium *S. aureus* Xen29 in various reporter media following 24 and 48 hours of incubation with several test bacteria.

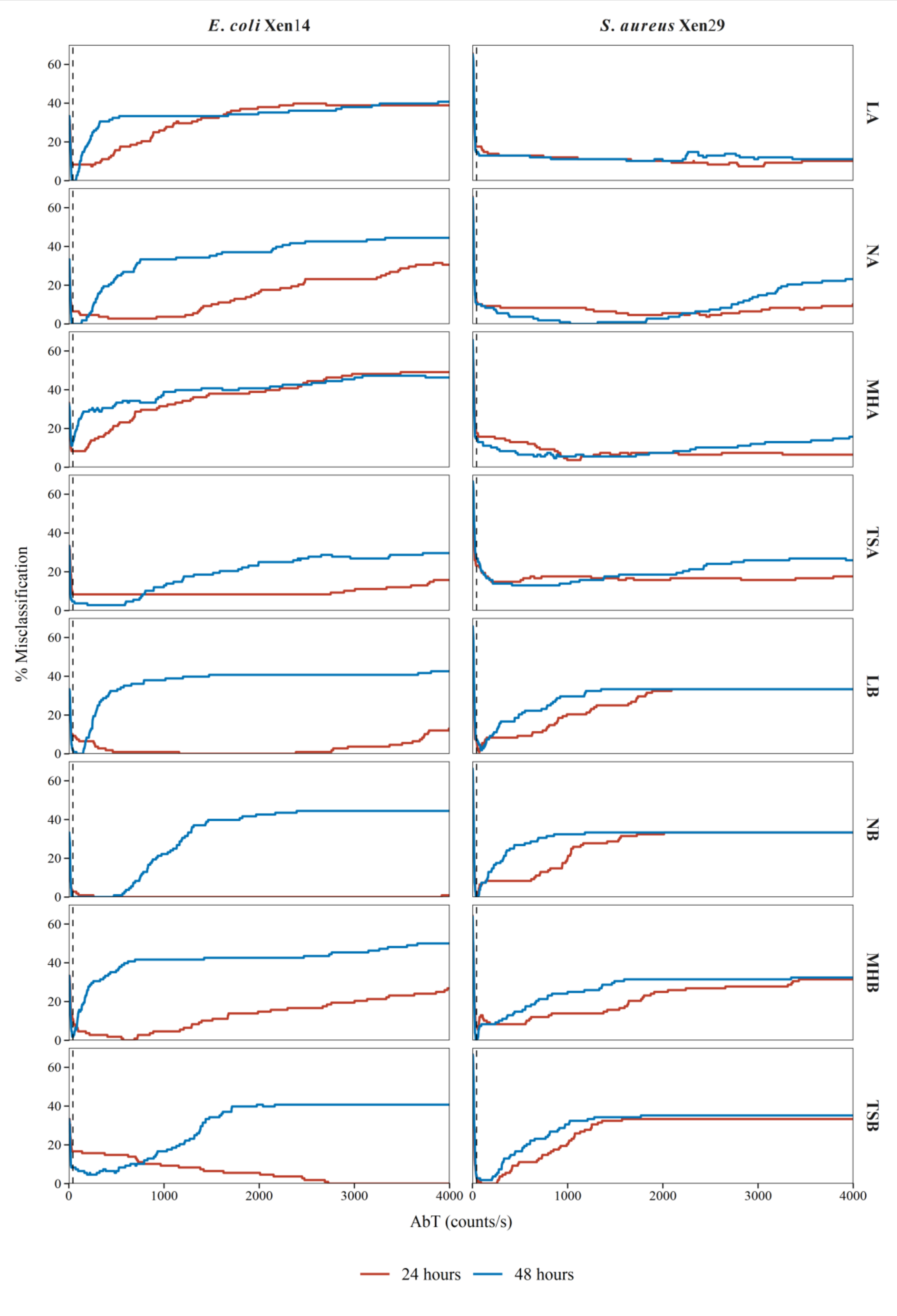

Figure S4. Misclassification of test bacterium activity towards the reporter bacteria *E. coli* Xen14 and *S. aureus* Xen29 at different AbT in various reporter media following 24 and 48 hours of incubation. Dashed line is indicative of the AbT set during the study at 43 counts/s.

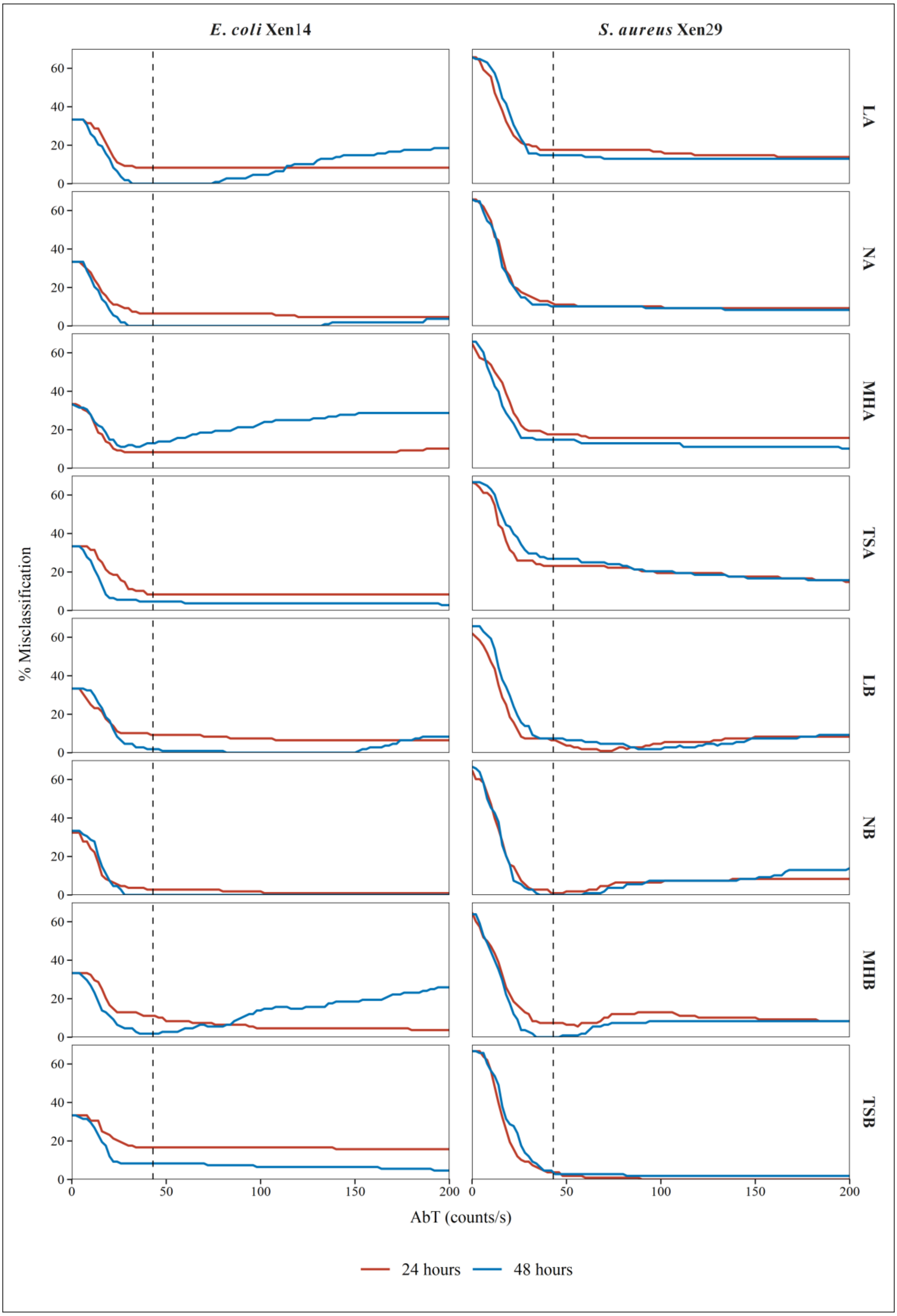

Figure S5. Misclassification of test bacterium activity towards the reporter bacteria *E. coli* Xen14 and *S. aureus* Xen29 at different AbT between 0 and 200 counts/s in various reporter media following 24 and 48 hours of incubation. Dash line is indicative of the AbT set during the study at 43 counts/s.
